# Relationship Between US Pediatric Resident Burnout and the Learning Environment

**DOI:** 10.1177/00099228251326652

**Published:** 2025-08-25

**Authors:** E. Rodriguez Lien, E. Zwemer, A. Schwartz, P. M. Wilson, JC. Babal, J. R. Serwint, K. Sieplinga, K. Donnelly, M. Nichols, M. Batra

**Affiliations:** 1 Department of Pediatrics, School of Medicine, University of Texas Medical Branch, Galveston, TX, USA; 2 Department of Pediatrics, School of Medicine, University of North Carolina at Chapel Hill, Chapel Hill, NC, USA; 3 Departments of Medical Education and Pediatrics, University of Illinois at Chicago, Chicago, IL, USA; 4 Division of Emergency Medicine, Department of Pediatrics, University of Cincinnati, College of Medicine, Cincinnati, OH, USA; 5 Department of Pediatrics, School of Medicine and Public Health, University of Wisconsin, Madison, WI, USA; 6 Department of Pediatrics, School of Medicine, Johns Hopkins University, Baltimore, MD, USA; 7 Department of Pediatrics, Helen DeVos Children’s Hospital, Corewell Health, Grand Rapids, MI, USA; 8 Department of Pediatrics, Inova Children’s Hospital, Falls Church, VA, USA; 9 Department of Pediatrics, Division of Emergency Medicine, University of Alabama at Birmingham, Birmingham, AL, USA; 10 Department of Pediatrics, School of Medicine, University of Washington, Seattle, WA, USA

**Keywords:** pediatrics, residency, burnout, well-being, learning environment

## Abstract

There is a paucity of literature evaluating the association between the pediatric residency learning environment (LE) and resident well-being. A cross-sectional study investigated the association between pediatric residents’ LE satisfaction and their burnout, and whether LE subcomponents influenced LE satisfaction. A total of 2043 (69%) residents representing 46 pediatric programs responded, indicating that 40% of participants met the burnout classification. Residents not meeting burnout classification reported greater mean LE satisfaction (4.4 vs 3.6, *P* < .001), LE collaboration (4.4 vs 4.1, *P* < .001), resident mentorship (3.7 vs 3.1, *P* < .001), and resident education (4.1 vs 3.5, *P* < .001) than their colleagues with burnout. Residents reporting greater LE collaboration (*B* = 0.62, 95% confidence interval (CI) = 0.57-0.67, *P* < .001), greater resident mentorship (*B* = 0.51, 95% CI = 0.48-0.55, *P* < .001), and greater resident education (*B* = 0.72, 95% CI = 0.68-0.75, *P* < .001) had higher LE satisfaction, controlling for individual/program characteristics and clustering. This study demonstrates associations between LE satisfaction and burnout from a national group of pediatric residents.

## Introduction

The Pediatric Resident Burnout-Resilience Study Consortium (PRB-RSC) Learning Environment (LE) Subcommittee conducted an exploratory, multicenter cross-sectional study with the Association of Pediatric Program Directors’ Longitudinal Educational Assessment Research Network (APPD LEARN)^1^ to assess the relationship between resident LE satisfaction with resident burnout and additional LE subcomponents. LE subcomponents included resident perceptions of LE collaboration, career mentorship, and the program’s commitment to education.

Resident burnout is a pervasive problem which has been shown to compromise patient safety and contribute to personal and professional deterioration.^2,3^ To date, research has focused mainly on factors that influence the individual trainee rather than system influences where the training occurs.^2,4,5^ The LE as an influencer of resident well-being has been recognized by the Accreditation Council of Graduate Medical Education (ACGME) with the creation of the Clinical Learning Environment Review (CLER) Program in 2012. The objective of CLER is to “improve how clinical sites engage resident and fellow physicians in learning to provide safe, high quality patient care,” with well-being being one of its 6 identified focus areas.^6^

The PRB-RSC was formed in 2015 to conduct pediatric residency well-being research.^3^ Since its inception, the PRB-RSC has conducted annual resident surveys to assess well-being parameters, including burnout. It previously reported preliminary results relating the LE to burnout, noting that higher LE satisfaction was associated with lower rates of burnout.^3^ The 2019 PRB-RSC survey expanded on the single question in the previous iteration to address specific LE factors including mistreatment, education, mentorship, and LE collaboration.^7^ It was the first attempt at evaluating whether a relationship exists between LE elements and resident burnout. The PRB-RSC LE Subcommittee was formed in 2019 to further evaluate these relationships.

While there is no universal LE definition, there has been an evolution in its conceptual framework. Most recently, Norquist et al^8^ suggested 6 LE “avenues,” including psychological, educational, sociocultural, architectural, digital, and diversity and inclusion. When reviewing literature outside of pediatrics, components of these avenues have been highlighted. Van Vendeloo, Lases, and Lee include collaboration, resident mentorship, and resident education as metrics of LE quality, representing the socio-cultural and educational avenues.^9-12^ A recent pediatrics study highlighted the relationship between resident mistreatment on perceived burnout, exemplifying the psychological avenue.^7^ However, the paucity of literature relating LE characteristics to pediatric resident burnout led our group to further investigate these relationships.

The primary objective of this study was to evaluate the relationship between LE satisfaction and resident burnout. The secondary objective was to evaluate how specific LE subcomponents influence resident LE satisfaction. We hypothesize pediatric residents reporting higher LE satisfaction will also report lower burnout, consistent previous preliminary data.^3^ Second, we hypothesize greater perceived LE collaboration, resident career mentorship, and resident education as a program priority will be associated with higher LE satisfaction.

## Methods

The PRB-RSC membership, overall design, participant eligibility and recruitment, and use of standard instruments through APPD LEARN have been previously described.^13,14^ This study is an extension of the PRB-RSC ongoing work since 2016 and complies with SQUIRE guidelines. The study was conducted in accordance with the Declaration of Helsinki. Institutional Review Board (IRB) approval was granted for each participating site prior to survey distribution. The IRB at Nationwide Children’s Hospital acted as the central IRB, whose review was accepted by all participating institutions’ IRBs (Ref. IRB15-00831, dated November 20, 2015). The central IRB determined that this research involved minimal risk and approved a waiver for informed consent.

Data extracted from the annual survey included resident demographics, program characteristics, results of the 2-item Maslach Burnout Inventory (MBI),^13^ LE satisfaction, and their perceptions of the LE components. Demographic information included age, sex, race, marital/partnered status, parental status, and program size (small <30, medium 30-60, large >60).

Pediatric residents from 46 US residency programs, including categorical and combined programs, responding to the 2019 PRB-RSC annual survey were included. There were no exclusion criteria. Consortium involvement and survey completion were voluntary. Residents submitted their electronic survey to APPD LEARN in de-identified form using a unique LEARN ID. At the conclusion of data collection, data were further encrypted to prevent resident identification by APPD LEARN or an individual institution.

Resident Burnout was determined by the 2-item burnout screen, which correlates with the 22-item MBI with 85% to 87% sensitivity and 84% to 85% specificity.^13^ Burnout was defined as “feeling burned out from my work” and/or “[becoming] callous to other people since [taking] this job” once weekly or more. Residents screening positive for burnout were classified as PBO (Positive Burnout), and those not meeting burnout criteria were classified as NBO (Negative Burnout).

Learning environment satisfaction and LE subcomponents queries in the survey were previously constructed by a team of medical education researchers, including a survey review expert (PW) for content validity. The group used an iterative process for survey development, and the final format was reviewed by the PRB-RSC steering committee. Learning environment satisfaction was assessed by asking residents, “Please rate your satisfaction with the overall learning environment in which you work,” on a 5-point Likert scale (very dissatisfied, somewhat dissatisfied, neutral, somewhat satisfied, very satisfied). Satisfied residents were defined as those who responded somewhat (4) or very satisfied (5) to this question.

Learning environment subcomponents were evaluated on a 5-point Likert scale, asking respondents to rate their agreement with corresponding statements, ranging from strongly disagree (1) to strongly agree (5). Resident perception of LE collaboration was assessed by asking respondents to rate their agreement with the statement, “I work in a collaborative rather than a competitive environment.” Resident perception of their program’s mentorship was assessed by asking respondents to rate their agreement with the statement, “Resident career mentoring is a high priority in my program.” Resident perception of their program’s commitment to education was assessed by asking respondents to rate their agreement with the statement, “Resident education is a high priority in my program.”

### Statistical Analysis

We analyzed the relationship between mean Likert scale scores for resident LE satisfaction and burnout classification using a mixed-effects logistic regression model. Burnout was defined as the outcome and LE satisfaction as the key predictor, along with a random effect of program to control for clustering within programs. We fit models including learner and program covariates to assess whether the effect of LE satisfaction persisted adjusting for demographics, including program size (small, medium, or large), resident gender, race, age (in years), marital status, and whether the resident had children. Given previously reported associations between mistreatment and higher levels of burnout, mistreatment was identified a priori as a potential confounder.^7^ Mistreatment was defined as answering “yes” to one or more experiences related to bullying, discrimination, sexual harassment, or physical violence within the past 12 months.

We used a set of similar mixed-effects linear models to investigate potential associations between LE satisfaction and individual LE subcomponents. These subcomponents were chosen a priori and based on previous literature evaluating LE quality.^9-12^ Each model included the subcomponent, demographic and mistreatment covariates, and a random effect of program to adjust for clustering.

## Results

Overall, 2958 residents were eligible for the study with 2046 residents responding and 2043 providing complete data, leading to a 69% response rate (Table 1). Residents represented 46 programs across the country with response rates by region ranging from 31% in the Southwest to 75% in the Midwest. Categorical pediatric residents represented 84.8% of participants (n = 1733), while Medicine-Pediatrics (n = 179) and other pediatric combined programs (n = 134) made up 15.2%. Reported White race was associated with a higher PBO classification than those not indicating White race. Medium-sized programs were less likely to have residents meeting the PBO classification, and Western and New England programs were more likely to have residents meeting the PBO classification.

**Table 1.** Demographics.

n = 2043	NBO^a^ (%) n = 1226	PBO^b^ (%) n = 817	*P* value
Sex			
Male, n = 601	378 (63)	223 (37)	0.08
Female, n = 1435	843 (59)	592 (41)	
Other, n = 7	5 (71)	2 (29)	
Age			
Mean (years)	29.4	29.6	0.51
Marital status			
Single/divorced, n = 842	494 (59)	348 (41)	0.24
Married/partnered, n = 1192	730 (61)	462 (39)	
Children			
No, n = 1671	995 (60)	676 (40)	0.47^c^
Yes, n = 306	189 (62)	117 (38)	
Missing, n = 66	42 (64)	24 (36)	
Race			
White, n = 1424	818 (57)	606 (43)	<0.001
Non-White, n = 619	408 (66)	211 (34)	
Program size			
Large, n = 1391	806 (58)	585 (42)	0.02
Medium, n = 598	388 (65)	210 (35)	
Small, n = 38	22 (58)	16 (42)	
Country region			
Mid-America, n = 424	266 (63)	158 (37)	<0.001
Mid-Atlantic, n = 260	153 (59)	107 (41)	
Midwest, n = 342	229 (67)	113 (33)	
New England, n = 100	49 (49)	51 (51)	
New York, n = 149	92 (62)	57 (38)	
Southeast, n = 317	195 (62)	122 (38)	
Southwest, n = 102	63 (62)	39 (38)	
West, n = 333	169 (51)	164 (49)	

a Residents not meeting burnout criteria (negative burnout); ^b^Residents meeting burnout criteria (positive burnout); ^c^Excluding missing responses.

### Unadjusted Associations With Burnout

Notably, 39.9% of residents met the burnout classification. The mean overall LE satisfaction was 4.0 (SD 1.0). Greater mean LE satisfaction was noted in residents classified as NBO compared with PBO (4.4 vs 3.6, *P* < .001) (Table 2). NBO residents were also significantly more likely to report greater mean LE collaboration (4.4 vs 4.1, *P* < .001), resident mentorship (3.7 vs 3.1, *P* < .001), and resident education (4.1 vs 3.5, *P* < .001).

**Table 2.** Outcomes.

Resident Burnout	Total	NBO^a^	PBO^b^	*P* value
LE Satisfaction, mean (SD)	4.0 (1.0)	4.4 (0.8)	3.6 (1.1)	<0.001
LE Collaboration, mean (SD)	4.3 (0.8)	4.4 (0.7)	4.2 (0.8)	<0.001
Resident Mentorship, mean (SD)	3.4 (1.0)	3.7 (0.9)	3.1 (1.1)	<0.001
Resident Education, mean (SD)	3.8 (0.9)	4.1 (0.8)	3.5 (1.0)	<0.001
LE^c^ Satisfaction Associations	*B* coefficient	95% CI	*P* value	
LE Collaboration	0.62	0.57-0.67	<0.001	
Resident Mentorship	0.51	0.48-0.55	<0.001	
Resident Education	0.72	0.68-0.75	<0.001	

a Residents not meeting burnout classification (negative burnout); ^b^Residents meeting burnout classification (positive burnout); ^c^Learning Environment.

### Adjusted Associations With Burnout

**Figure 1.**
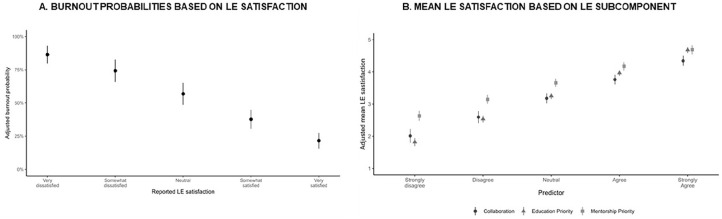
(A) Burnout probabilities based on LE satisfaction. (B) Mean LE satisfaction based on LE subcomponent.

In a multivariate logistic regression controlling for clustering within programs, LE satisfaction was associated with lower odds of PBO classification (odds ratio (OR) 0.44, 95% confidence interval (CI) 0.37-0.53, *P* < .001; Figure 1A). Higher resident perception of program mentorship had a statistically significant lower odds of PBO classification (OR 0.88, 95% CI 0.77-1.0, *P* = .05) but perceived LE collaboration (OR 1.0, 95% CI 0.85-1.18, *P* = .99) and resident education (OR 1.02, 95% CI 0.86-1.21, *P* = .84) did not. Additionally, medium (vs large) programs (OR 0.74, CI 0.55-0.98, *P* = .04), older resident age (OR 0.94, CI 0.90-0.98, *P* = .01), and non-White race (OR 0.60, CI 0.47-0.76, *P* < .001) were associated with lower odds of PBO classification.

In the final regression excluding program size but including program mean LE satisfaction (ie, mean LE satisfaction reported among residents at each residency site) as a covariate, program mean LE satisfaction had no significant independent association with burnout.

After controlling for demographic variables and mistreatment, greater perceived LE collaboration (*B* = 0.63, 95% CI 0.57-0.67, *P* < .001), greater perceived resident mentorship (*B* = 0.51, 95% CI 0.48-0.55, *P* < .001), and greater perceived resident education (*B* = 0.72, 95% CI 0.68-0.75, *P* < .001) was associated with greater LE satisfaction (Figure 1B and Table 2).

## Discussion

Our study demonstrates an inverse association between PBO classification and LE satisfaction within US pediatric residents and offers insight into specific LE subcomponents that programs can focus their improvement efforts. These findings are highly generalizable as this dataset represents the largest pediatric program collaboration nationally. This study is the first step in identifying areas for LE intervention.

The overall resident burnout rate of 40% in our study was lower than previously published work by the PRB-RSC with rates of 55%, 53%, and 52% for the 2016, 2017, and 2018 cohorts, respectively. We hypothesize this is related to increased efforts by programs to address resident well-being (including burnout) after the 2017 ACGME mandates in the program common requirements.^15^ Our findings demonstrated a higher mean LE satisfaction in residents who did not meet burnout classification. classify as burned out. Van Vendeloo and colleagues also noted an inverse burnout-LE relationship among Dutch residents spanning 33 specialties in 2018, with similar burnout rates.^9,10^ Our study also demonstrated residents reporting burnout were more likely to report LE dissatisfaction, even if mean program LE satisfaction was high. This suggests that residents experiencing burnout are more likely to perceive the LE negatively regardless of the overall milieu. This may be related to individual factors given their burnout status and general dissatisfaction or may indicate some aberrant system component critically affecting these residents. It is important to note that burnout is just one component of resident well-being. Emerging literature notes positive associations between the LE climate and residents’ work-related well-being.^11^

To date, key drivers impacting resident LE satisfaction have not been well delineated. Previous studies evaluating the LE have used resident perceptions of collaboration, mentorship, and education as a measure of LE quality.^3,9,10,12^ Our study noted a positive association between resident perception of LE collaboration, resident mentorship, and resident education with LE satisfaction. The provision of academic resources has also been shown to correlate with improved LE perceptions among surgical residents,^12^ which may explain the variability noted in burnout rates based on program size.

Limitations of this study include the reliance on self-reported data, though validated scales were used whenever possible. LE collaboration, resident mentorship, and resident education questions were non-validated and exploratory in nature and based on resident perceptions. Data are specific to pediatric and medicine/pediatric residents and may not be generalizable to other disciplines. The interpreted data was pre-pandemic; thus, current rates of burnout may differ, however, the association between burnout and LE has been previously identified in other disciplines^7,9-12^ as well. Associations do not provide causality and further work is needed to delineate root causes. Finally, due to the lack of an agreed-upon LE definition, this broad term is subject to interpretation bias. While Nordquist has provided a framework to describe the LE,^8^ future studies should explore resident LE component perceptions as our study only included 2 of the avenues described.

This is an exploratory study investigating associations that could provide a better understanding of the LE-burnout relationship and may influence how programs approach well-being initiatives. While residents reporting burnout are more likely to report LE dissatisfaction even if mean program LE satisfaction is high, this should not preclude program leadership from evaluating LE components and inquire about resident LE perceptions. Implications of this work include the need to explore the root causes of the LE satisfaction-burnout relationship, define key components of the LE, evaluate the extent to which each LE component may impact well-being, and identify what contributes to opinions of LE collaboration, mentorship, and education.

## Conclusions

This study demonstrates the inverse relationship between LE satisfaction and pediatric resident burnout. Furthermore, resident perceptions of greater LE collaboration, resident career mentorship, and resident education were associated with higher LE satisfaction.

## Author Contributions

All authors provided substantial contributions to the conception or design of the work, data acquisition, analysis, or interpretation, drafting the work, or revising it critically for important intellectual content, approving the final version, and agreeing to be accountable for all aspects of the work.
